# Acute Changes in Thyroid Hormone Levels among Thai Pesticide Sprayers

**DOI:** 10.3390/toxics9010016

**Published:** 2021-01-19

**Authors:** Pornpimol Kongtip, Noppanun Nankongnab, Ritthirong Pundee, Nichcha Kallayanatham, Sumate Pengpumkiat, Jutamanee Chungcharoen, Chavisa Phommalachai, Pajaree Konthonbut, Nattagorn Choochouy, Preecha Sowanthip, Phanthawee Khangkhun, Jutharak Yimsabai, Susan Woskie

**Affiliations:** 1 Department of Occupational Health and Safety, Faculty of Public Health, Mahidol University, 420/1 Rajvidhi Road, Bangkok 10400, Thailand; noppanun.nan@mahidol.ac.th (N.N.); nichcha.kal@gmail.com (N.K.); sumate.pen@mahidol.ac.th (S.P.); yiesipttt@gmail.com (J.C.); chawisa.ph8@gmail.com (C.P.); pajaree.kon@mahidol.ac.th (P.K.); 2 Center of Excellence on Environmental Health and Toxicology, EHT, Bangkok 10400, Thailand; preecha.sow@mahidol.ac.th; 3 Nakhonsawan Campus, Mahidol University, Nakhonsawan 60130, Thailand; rtg.pun@gmail.com; 4 Faculty of Public Health, Thammasat University Lampang Campus, Lampang 52190, Thailand; n.choochouy@gmail.com; 5 Bureau of Elderly Health, Department of Health, Ministry of Public Health, Nonthaburi 11000, Thailand; phanthawee.kha@gmail.com; 6 Department of Medical Technology and Clinical Pathology, Buddhachinaraj Phitsanulok Hospital, 90 Sithamma Traipidok Road, Muang, Phitsanulok 65000, Thailand; jutharak@gmail.com; 7 Department of Public Health, University of Massachusetts Lowell, One University Ave, Lowell, MA 01854-2867, USA; Susan_Woskie@uml.edu

**Keywords:** cypermethrin, paraquat, glyphosate, chlorpyrifos, thyroid hormones, acute exposure, farmers, pesticides, endocrine disrupter

## Abstract

The objective of this study was to investigate the relationship of acute pesticide exposures and acute changes in thyroid hormones among Thai farmers. We recruited 78 farmers, who were scheduled to spray insecticides (chlorpyrifos and/or cypermethrin) or herbicides (paraquat and/or glyphosate). On the day before spraying, farmers collected their first morning void urine and went for blood collection. On the spray day, urine samples were collected at end of the spraying event and they were interviewed with questionnaires. The next morning, the first morning void urine and blood samples were collected. Blood samples were analyzed for thyroid hormones. Urine samples were analyzed for the metabolites of the pesticide sprayed. The results showed that the thyroid hormones, free triiodothyronine (FT3) and total triiodothyronine (T3) were significantly reduced as urinary chlorpyrifos metabolite increased the day after spraying. Total thyroxine (T4) significantly increased as cypermethrin metabolites increased the day after spraying. T4 significantly increased as urinary glyphosate levels increased; however, FT3 and T3 decreased significantly as urinary paraquat levels increased the day after spraying. These findings suggest that acute exposures to the pesticides chlorpyrifos, cypermethrin, paraquat and glyphosate can produce acute effects on the hypothalamic–pituitary–thyroid (HPT) axis, acutely altering thyroid hormone levels.

## 1. Introduction

Over 120 pesticides have been listed as potential endocrine-disrupting chemicals (EDCs) [1,2,3]. Many insecticides are considered EDCs, including organophosphates (OPs) such as chlorpyrifos-methyl and malathion, and pyrethroids such as cypermethrin, deltamethrin and permethrin. Some herbicides are also considered EDCs, including glyphosate and atrazine [1]. EDCs can impact the hypothalamic–pituitary–thyroid (HPT) axis and alter thyroid hormone levels by disrupting central regulation, iodine uptake, production and distribution of thyroid hormones, or binding of the thyroid hormones to membrane transporters or receptors [4,5,6].

Currently, OPs remain one of the most commonly used pesticides [7]. Several epidemiologic studies have established an association between OPs and alterations of thyroid hormone levels [8,9]. A limited number of studies have examined whether ever using OP pesticides was associated with hypothyroidism [9,10,11], or whether use in the past growing season was related to alterations in thyroid hormone levels [12,13,14]. Experimental animal studies have also shown that organophosphate (OP) disturb thyroid gland function [15,16]. Whether the thyroid effects are caused by the parent OPs or the DAP metabolites is not known [7]. When humans are exposed to the OP, it is metabolized into the specific metabolite 3,5,6-trichloro-2-pyridinol (TCP) and the nonspecific OP metabolites diethyl phosphate (DEP) and diethyl thiophosphate (DETP) [17,18].

Synthetic pyrethroids are insecticides that have been introduced over the past two decades for agricultural and domestic use [19]. Pyrethroids are widely used in agriculture, animal husbandry and in homes to control insect pests (ants, cockroaches, mosquitos) [20,21]. Cypermethrin, a synthetic pyrethroid, was found to induce neurotoxicity in animal studies through free radical formation, reduction in the antioxidant defense mechanism, and inhibition of acetylcholinesterase (AChE) activity [22]. Pyrethroids also exhibit endocrine disrupting properties [1,23,24], and may impact fertility [25]. Human studies of thyroid hormone levels related to pyrethroid exposure have been inconclusive to date, in part due to low exposures [24] or mixed pesticide exposures [26].

Glyphosate herbicides are widely used in agricultural areas in Thailand. In plants, glyphosate acts through inhibition of the shikimate metabolic pathway [27]. Acute intoxication of humans by glyphosate can cause hypotension, tachycardia, renal failure, respiratory distress, metabolic acidosis, and electrolyte imbalances [28]. Glyphosate is not readily metabolized in the human body, and thus the parent compound can be measured in urine. There is mixed evidence regarding the impact of glyphosate on the thyroid. The U.S. Agricultural Health study has reported that among pesticide applicators, self-reported use of glyphosate is associated with hypothyroidism [9]. However, subsequent studies in the same cohort did not confirm this finding [11,29].

For paraquat, acute poisoning can result in lung damage, pneumonitis and lung fibrosis, renal and liver injury, acute renal failure, respiratory failure, and mucosal injury. Due to its high acute toxicity and adverse effects on human health, paraquat is now banned in over 50 countries including the 27 countries of the European Union, Cambodia, China, Vietnam, and Thailand [30,31,32]. Information on the effect of paraquat on thyroid function is limited to agricultural populations in Brazil [33].

The thyroid plays an important role in metabolism, thermogenesis, immunity, and aging and changes in thyroid hormone production with age is associated with changes in other organs and systems [34]. Franceschi et al. proposed the concept of “thyroid biography” to cover the combination of factors that impact thyroid function over the lifespan, including genetics, exposure to endocrine disruptors and lifestyle habits [34]. Changes in thyroid status with age are in turn associated with cognitive and functional status and mortality [35]. We have previously shown in the larger longitudinal cohort, from which the farmers in this study were recruited, that after adjusting for covariates, thyroid hormone levels of conventional pesticide using farmers were significantly higher than those of organic farmers. Several specific herbicides had a significant relationship between the amount applied and an increase in thyroid hormone levels [12], and increasing the cumulative number of spray days of pesticides significantly increases the level of thyroid stimulating hormone (TSH) and free triiodothyronine (FT3) [13]. These farmers also had significantly higher abnormal metabolic biomarkers, body mass index (BMI), waist circumference, % body fat, triglycerides, total cholesterol, and low-density lipoprotein (LDL) values) compared to the organic farmers [36], and there was a significant association between the number of days of pesticide spraying and levels of cholesterol, high-density lipoprotein (HDL), LDL, blood pressure and BMI in the longitudinal cohort [37]. These findings support the proposal that pesticides may function as endocrine disrupters, in part through the HPT axis, increasing the risk for abnormal metabolic biomarkers which are linked to the development of long term metabolic disease (Cardiovacular disease, stroke, diabetes). Nevertheless, how repeated acute exposures to pesticides can result in these chronic health effects has not been clearly demonstrated.

Like our previous work, most of the human studies related to the effects of pesticides on thyroid hormones refer to longer term chronic exposures and often involve exposures to many types of pesticides including insecticides, herbicides, and fungicides. We did find one study in India that examined the effect of acute poisoning from organophosphate compounds on the thyroid profile of individuals. The results showed that T4 was significantly higher in the acute poisoning phase and went down after a one-month recovery, while TSH and T3 were significantly reduced in the acute poisoning phase and then returned to normal after recovery [38].

This study was designed to investigate the relationship of acute pesticide exposures and acute changes in thyroid hormones among Thai farmers. We measured farmer’s exposure (change in metabolite levels) to chlorpyrifos or cypermethrin insecticides, paraquat or glyphosate herbicides from the day before to the day after spraying along with changes in levels of thyroid hormones from the day before to the day after spraying.

## 2. Materials and Methods

### 2.1. Study Population and Data Collection

Data collection for this study (November 2016 to January 2019), was approved by the Ethical Review Committee for Human Research, Faculty of Public Health, Mahidol University (Approval No. MUPH 2015-146, date of approval: (28 August 2015). Informed consent was obtained from all participants. From our larger longitudinal study of conventional (pesticide using) and organic farmers (*n* = 438), we recruited seventy-eight sugarcane farmers from Khao Thong Subdistrict, Phayuha Khiri District in Nakornsawan province, in the upper central area in Thailand. We selected one farmer who sprayed pesticides in each family. They were male or female over 18 years old, free of a current diagnosis of diabetes, high blood pressure, and thyroid or heart disease. The field staff set an appointment with the farmers when they planned to spray chlorpyrifos, cypermethrin, paraquat or glyphosate in their fields. Most of the farmers sprayed one pesticide at a time and some participated on multiple days when they sprayed other targeted pesticides. Some of the farmers mixed other chemicals in with the targeted pesticides. The farmers spraying one chemical per day were 91.7% for glyphosate, 83.3% for cypermethrin, 45.1% for chlorpyrifos and 33.3% for paraquat; the rest of each group mixed with other chemicals such as acetochlor, 2,4 D, amethrin, diuron, etc. They sometimes mixed with 1–2 pesticides to save time and cost of spraying. Before the spraying day, urine samples were collected at waking, as first morning void and stored in an insulated carrier with ice to bring to the nearby clinic, where blood samples were collected between 7–9 a.m., and subjects were interviewed. The questionnaire covered home and demographic information, agricultural activities, history of pesticide mixing and spraying, use of personal protective equipment, acute health symptoms after spraying pesticides and a measure of self-reported stress that covered sleeping problems, reduced concentration, irritation, boredom, and lack of interest in meeting people. On the spray day, the field staff observed the spraying and interviewed the subject about the amount of pesticide used and the subject provided a urine sample at the end of the spraying event. The next day, the subject collected a first morning void urine and again came to the clinic between 7–9 a.m. for blood collection. The blood samples were used for thyroid hormone measurements. Urine samples were analyzed for biomarkers of exposure (metabolites of the sprayed pesticides).

### 2.2. Blood Analysis

Sera extracted from blood samples in non-heparinized vacutainer tubes were stored at −20 °C until analysis. All samples were analyzed at Buddhachinaraj Hospital, the regional medical center in Phitsanulok province using standard clinical laboratory methods. Analysis of thyroid hormones using a paramagnetic particle, chemiluminescent immunoassay for the quantitative determination of TSH, FT3, free thyroxine (FT4), total triiodothyronine (T3), and total thyroxine (T4) in human serum using the UniCel DxI 800 Access Immunoassay System, Beckman Coulter (Atlanta, GA, USA) [12]. The limit of detection was 0.005 μIU/mL for TSH, 0.09 ng/dL for FT3, 0.15 ng/dL for FT4, 0.01 μg/dL for T3 and 0.5 μg/dL for T4.

### 2.3. Urine Sample Analysis

Urine samples from chlorpyrifos-spraying farmers were analyzed for TCP (3,5,6-trichloro-2-pyridinol), the specific metabolite of chlorpyrifos using gas chromatography-mass spectrometry (GC-MS), the detection limit of TCP in urine was 1 ng/mL [39].

The analysis of the non-specific OP metabolites of chlorpyrifos diethylphosphate (DEP) and diethylthiophosphate (DETP), used the GC-MS method of Prapamontol et al., (2014) [40] except that the derivatization with 2,3,4,5,6-Pentafluorobenzyl bromide was conducted at 80 °C for 1.5 h. The recovery of a mixed DEP and DETP standard of 250 and 750 ng/mL ranged from 97–101% with relative standard deviation (RSD) of less than 10%. The detection limit of the DEP and DETP was 4.53 and 5.12 ng/mL, respectively. The results presented used total DEP(TDEP) = DEP + DETP.

Urine samples from cypermethrin-spraying farmers were analyzed for 3-phenoxybenzoic acid (3PBA) and *cis*-3-(2,2-dichlorovinyl)-2,2-dimethylcyclopropane-1-carboxylic acid, *trans*-3-(2,2-dichlorovinyl)-2,2-dimethylcyclopropane-1-carboxylic ac-id (cis, *trans*-DCCA) using GC-MS, following the method of Singleton et al. (2014) [41]. The recovery of 3PBA and cis, *trans*-DCCA concentrations of 25, 100 and 150 ng/mL ranged from 97.86–101.04% with RSD less than 6%. The results were presented as total cypermethrin metabolites(Tcyper) = 3BPA + *cis*, *trans*-DCCA.

Urine samples from paraquat- and glyphosate-spraying farmers were analyzed for paraquat [42] and glyphosate [43] using high-pressure liquid chromatography with fluorescent detector. The recovery of urinary paraquat for between-day was 95.83% and 96.52% and RSD was 3.13% and 4.04% for 30 and 80 ng/mL paraquat in urine, respectively with the detection limit of 1 ng/mL. The accuracy of glyphosate in urine was 76.88 and 98.40% and RSD was 3.26 and 2.85 at concentrations of 20 and 100 ng/mL with the detection limit of 1 ng/mL. For concentrations below the detection limit, we substituted the detection limit by dividing the detection with 2 for GSD <3 or the detection limit divided by two for GSD ≥3 [44]. All urinary metabolites were creatinine corrected (per gram creatinine). The creatinine in urine was analyzed using an enzymatic method with a linear concentration range of 1–500 mg/dL and a detection limit 0.16 mg/dL [45].

### 2.4. Statistical Analysis

All statistical analyses were done by SPSS for Windows, version 23 (IBM Thailand Co., Ltd., Bangkok, Thailand). Descriptive analysis of the demographic characteristics was done using Chi Square, Fisher’s exact test and independent *t*-tests. Due to the skewed distribution of the thyroid hormone and pesticide urinary metabolite data, the natural logarithm was used in all analyses. The natural log values for the urinary metabolite concentrations for the day before spraying, end of spraying task and the next day after spraying were compared using a repeated measure ANOVA. Natural log thyroid levels on the day before and the next day after spraying were compared using a paired *t* test. The model for change in thyroid hormone levels was performed using a generalized linear model. For each thyroid hormone, we developed a multivariable generalized linear model for change in the thyroid hormone level (logged morning after spraying—logged morning before spraying) as a function of the change in the urinary metabolite level for a specific pesticide (logged morning after spraying—logged morning before spraying). Additional covariates for each model were considered based on their significance in univariate analyses. In our previous cross-sectional study, we included gender, current smoking, current alcohol use, insecticide use at home in the past year, triglyceride levels and any stress symptoms in the past 2–4 weeks [12], so we examined these covariates in our univariate models. Others have also included socioeconomic status [46,47] in their work, so we also examined education and debt status in univariate models.

## 3. Results

The average age of farmers was 49.6 years and they were mainly male (74.4%) (Table 1).

Most had graduated from high school (54.5%); 83.8% were married and 46.2% were in debt. More than half had a second job (51.3%) such as working in a hair salon, vehicle repair shop, food stand, grocery store, or construction site. Most reported drinking alcohol (66.7%), though few were current smokers (19.2%). Most reported having symptoms of stress (76.9%) in the past 2–4 weeks (Table 2). These farmers grew sugarcane (98.7%) and rice (83.3%), lived near (<1 km) their farmland (94.9%) and almost all (98.7%) used pesticides in their home.

For chlorpyrifos-spraying farmers, there was 100% detection for all time periods for urinary TCP. The log(e) of urinary TCP concentrations at the end of spraying and the next day after spraying were significantly higher from the day before spraying, and the urinary TCP concentrations at the end of the spraying event was also significantly lower than the next day after spraying (Table 3). There was also 100% detection for all time periods for urinary DEP and DETP, the non-specific OP metabolites of chlorpyrifos. The log(e) of urinary TDEP concentrations at the end of spraying and the next day after spraying were not significantly different from those the day before spraying, although the urinary TDEP concentrations at the end of the spraying event and the next day after spraying were significantly different (Table 3).

For cypermethrin-spraying farmers, there was 100% detection for all farmers at all time periods for 3PBA and TCyper, however urinary *cis*, *trans*-DCCA concentrations were detected in 68.1% (day before), 83% (after spraying) and 74.5% (next day) of the farmers. The log(e) of urinary 3PBA concentrations at the end of spraying and the next day after spraying were significantly higher than those the day before spraying, but the urinary 3PBA concentrations at the end of the spraying event and the next day after spraying were not significantly different. The log(e) of the urinary *cis*, *trans*-DCCA concentrations at the end of spraying and the next day after spraying were significantly higher than those the day before spraying, but the urinary 3PBA concentrations at the end of the spraying event and the next day after spraying were not significantly different. The log(e) of the urinary TCyper levels had a similar pattern to that of 3PBA. 

Urinary paraquat was detected in 62.7% (day before), 94.1% (after spraying) and 76.5% (next day) of the farmers (Table 4). The log(e) of urinary paraquat concentrations at the end of spraying and the next day after spraying were significantly higher than those the day before spraying, and the urinary paraquat concentrations at the end of the spraying event were also significantly higher than the concentrations the next day after spraying.

Urinary glyphosate was detected in 100% of the farmers in all time periods. The log(e) of the urinary glyphosate concentrations at the end of spraying and the next day after spraying were significantly higher than those the day before spraying, but the urinary glyphosate concentrations at the end of the spraying event and the next day after spraying were not significantly different.

With regard to thyroid hormones, the thyroid hormone levels on the day after spraying chlorpyrifos were significantly higher for FT4 and borderline higher for FT3, but the change in thyroid hormone levels from the day before to the day after spraying cypermethrin, paraquat or glyphosate were not significantly different (Table 5 and Table 6). We did not get different results for the simple paired analysis of the change in thyroid hormone levels for cypermethrin, paraquat or glyphosate when stratified by gender. However, for chlorpyrifos male farmers showed significantly higher FT4 and FT3 the day after spraying (*p* < 0.001 and *p* = 0.047, *n* = 37), while for female farmers there was only a borderline significant increase for T3 (*p* = 0.050, *n* = 14).

The models for change in thyroid hormone levels as a function of the change in urinary metabolite levels when spraying each specific pesticide showed that FT3 and T3, were significantly reduced as a function of the change per one µg/g creatinine LnTCP (Table 7). For cypermethrin-spraying farmers, there was a significant increase in T4 as a function of the change per one nmol/g creatinine LnDCCA (Table 8). For the herbicide paraquat, FT3 and T3 were significantly reduced as a function of the change per one µg/g creatinine LnParaquat (Table 9). However, the herbicide glyphosate showed a significant increase in T4 as a function of the change per one µg/g creatinine LnGlyphosate.

## 4. Discussion

The average age of farmers was almost 50 years of age and over half (54%) graduated from high school. Most of them were male (74%). Pesticide spraying was performed using a backpack sprayer which can weigh over 25 pounds before filling with pesticide solution. This may have led to largely male participants in this study. Farmers in Lao, Cambodia and Vietnam, were also mostly male and fewer had graduated from high school (29%), although they were generally of a similar age as the Thai farmers (averaging 45 years old for Lao and Cambodia and 50 for Vietnam) [48]. In this study, 19.2% of the farmers smoked and 66.7% drank alcoholic beverages, which is less than that reported for tobacco farmers in Brazil, 29.4% of whom smoked and 81.8% of whom drank alcohol if male. [49].

### 4.1. Changes in Urinary Pesticide Levels after Spraying

The GM of the urinary metabolite for chlorpyrifos (TCP) showed a significant increase from pre-spraying to after the spraying event and also the next morning after spraying. This suggests that chlorpyrifos is metabolized quickly, but because the half-life of TCP is ~27 h [50,51], it is still found in urine the next day. The TCP levels reported here for the day after spraying (GM 14.1 ug/g creatinine) are lower than a study in Vietnam that found a median urinary TCP of 45.7 µg/g creatinine 24 h after pesticide application [52]. We believe this is due to the shorter duration of spraying in the current study (average 38 min with range 7–120 min) compared to 5.2 h (range 3–7.5 h) in the Vietnamese study using similar motorized backpack spraying equipment. With regards to non-specific organophosphate (OP) metabolites of chlorpyrifos, the GM of TDEP was increased non-significantly from 458 nmole/g creatinine on the pre-spraying day to 541 nmole/g creatinine on the day after spraying. The elimination half-life of organophosphates was 15.5 h with oral route and 30 h with dermal route, so it is possible that metabolism to the non-specific metabolites was still occurring and if measurements were made later on in the day after spraying levels would have been significantly higher than the pre-spraying day [53].

All urinary Tcyper, 3PBA and DCCA levels at the end of spraying event were significantly increased from the pre-spraying day, but they were not significantly different with the next day after spraying. This may be due to the short elimination half-life of 3BPA (5.7 h) and DCCA (4.5–5.4 h) [54]. For cypermethrin-spraying farmers, the urinary 3PBA and DCCA are not specific metabolites for cypermethrin, so pre-spraying samples may reflect wider use of pyrethroids. All farmers used insecticides in their home and most of the insecticides used at home contained pyrethroid [54,55]. The 3PBA is a metabolite of cypermethrin as well as cyhalothrin, deltamethrin, esfenvalerate, fenpropathrin, permethrin, phenothrin [56]. The DCCA is a metabolite of cypermethrin as well as cyfluthrin and permethrin [56]. Panuwet et al. (2004) [57] reported no significant difference of urinary Tcyper, 3PBA and DCCA levels in four groups of farmers including cut-flower, vegetable, cut-flower and vegetable, pesticide free crop, since farmers who applied pesticide to their crops and farmers who did not were equally exposed to cypermethrin, permethrin, cyfluthrin and other pyrethroids.

The detection frequency of urinary paraquat was 62.7% on the day before spraying, 94.1% after the spraying event and 76.5 % in the morning after spraying. This was similar to the pattern seen in a study by Lee et al. (2009) [58], showing the detection frequency was 16.2%, 52.9% and 36.1% on the before-spraying day, on the spray day and the next day. We found a significant increase in paraquat levels from pre-spraying to after the spray event, which may reflect the quick distribution half-life of paraquat (5 h) [59]. However, we also saw a significant drop in the paraquat levels from after spraying till the next morning which was not expected due to paraquat’s slow elimination half-life of 84 h [59]. This current study may have found levels of urinary paraquat on the day before spraying day because sugarcane farmers work in their fields almost every day and may be exposed to paraquat through their agricultural activities, since paraquat has a long half-life in soil (up to 20 years) [31,60].

For urinary glyphosate, we found urinary glyphosate in 100% of urine samples collected. Acquavella et al. (2004) [61], detected a lower percentage of detectable urinary glyphosate in U.S. farmers on the pre-spraying day, spraying day and day after spraying, 15, 60 and 48% respectively. The high detectable levels pre-spraying may be due to the intensive use of glyphosate resulting in contamination of the environment. The half-life of glyphosate in soil is few days to two or three months [62]. In our study, the GM of urinary glyphosate increased significantly from pre-spraying to after spraying but the next morning, although higher, was it not significantly different from the end of spraying concentration. This may be attributable to the short elimination half-life of glyphosate; 4 and 17 hr in male and female [63]. Connolly et al. [63] found that the GM of urinary glyphosate on the day of spraying (1.2 μg/L) was significantly higher than that on the pre-spraying day (0.7 μg/L), but the GM of urinary glyphosate on the next day after spraying (0.8 μg/L) was not significantly higher than that on the pre-spraying day. In addition, in the Connolly et al. (2018) [64] study, the sprayers wore coverall suits, full-face respirators and gloves.

### 4.2. Changes in Thyroid Hormone Levels after Spraying

We found that the simple paired analysis of FT4 was significantly higher the morning after spraying chlorpyrifos and that FT3 increased with borderline significance. However, in models of thyroid level change that incorporated the measured change in metabolite levels and covariates, we found that an increase of one µg/g creatinine urinary TCP from before to after spraying significantly reduced the thyroid hormone levels of FT3 and T3 in the sprayers. We could not find research with a similar study design, but a study in the freshwater fish *H. fossilis* showed that exposure to 0.284 ppm chlorpyrifos for 30 days decreased serum T3, T4 and TSH significantly compared to the normal control group [65]. A study in rats found no statistically significant change in TSH, but a significant decrease in T3 and T4 among rats treated orally (6.75 mg/kg) for 30 days with chlorpyrifos, compared to the control group [66]. In models that incorporated the measured change in metabolite levels and covariates, we did not find a significant relationship for TDEP. However, a study of chronic DEP exposure of rats compared with a control group resulted in a significant decrease in serum TSH and FT3, but T3 and FT4 levels increased significantly in a dose-dependent manner [7].

The current study found that for those spraying cypermethrin, there were no significant changes in the simple paired analysis of thyroid hormone levels before and the morning after spraying. However, in models of thyroid level change that incorporated the measured change in metabolite levels and covariates, we found that an increase in DCCA was associated with a significant increase in T4. Zhang et al. (2013) [24] found that 3-PBA was not a significant predictor of serum thyroid hormones, FT4 and TSH and thyroid binding globulin in pregnant women. Male rats exposed to the pyrethroid fenvalerate (100–200 mg/kg) had significantly increased circulating thyroid hormone levels of T3 and T4. In lizards orally exposed to the pyrethroid lambda-cyhalothrin (LCT) for 21 days, both thyroid hormones, T3 and T4 were significantly increased compared to the control group [67]. When embryonic zebrafish were exposed to the pyrethroids bifenthrin or λ-cyhalothrin for 72 h; T4 hormone was significantly decreased while T3 hormone was significantly increased in the bifenthrin exposed group, whereas both T4 and T3 levels were significantly increased in the λ-cyhalothrin exposed group [68]. However, a human study of 18 workers exposed to pyrethroids in a pyrethroid insecticide manufacturing company found significantly reduced T3 and T4 and increased TSH in exposed workers compared to the control group [69], and a study of Brazilian agricultural workers who reported recent use of lambda-cyhalothrin (pyrethroid) had reduced total T4 [33].

This study did not find any significant change in the simple paired analysis of thyroid hormone levels from the day before to the day after spraying paraquat. However, in models of thyroid level change that incorporated the measured change in metabolite levels and covariates, we found that an increase in urinary paraquat from the day before to the day after spraying significantly reduced the T3 and FT3 hormones. For paraquat, US based studies found an association between ever use of paraquat and risk of hypothyroidism in women but not men [10,11]. Recent use of paraquat among Brazilian agricultural workers was associated with reduced FT3 [33].

For glyphosate spraying, we did not find any significant change in the simple paired analysis of thyroid hormone levels from the day before to the day after spraying. However, in models of thyroid level change that incorporated the measured change in metabolite levels and covariates, we found that an increase in urinary glyphosate from the day before to the day after spraying significantly increased the T4 hormone levels. A study of male offspring of pregnant Wistar rats exposed to glyphosate from gestation day 18 to post-natal day 5 found that at post-natal day 90, the offspring showed decreased TSH and no change in T3 and T4 compared to the control groups [70]. A cohort study of licensed pesticide applicators in North Carolina and Iowa with 35,150 participants showed that self-reported use of glyphosate was associated with increased risk of hypothyroidism [9] but it was not consistent with results reported by Goldner et al. (2013) [11] and Lerro et al. (2018) [29].

The findings of the simple paired analysis of thyroid hormone samples taken the morning before spraying and the next morning after spraying in some cases suggested changes in hormone levels in different directions (increasing vs decreasing) when compared to the results of the models that looked at change in hormone levels as a function of change in the measured change in pesticide metabolite levels. This could be because the models, that use metabolite levels, account for differences in the amount and length of spraying as well as use of personal protective equipment to limit exposure and personal hygiene practices to reduce dermal uptake. The simple before and after comparisons do not account for these differences or any individual differences in the ability to metabolize and excrete these pesticides. For example, polymorphisms in the genotype of PON-1, an enzyme that metabolizes OP pesticides, have been shown to directly impact susceptibility to OP toxicity [71]. In addition, unlike the simple paired comparison, the models adjusted for the impact of other potential confounders and covariates such as age, gender, smoking, stress etc.

Limitations of this study include the chronic pesticide exposures experienced by this cohort, which included farmers that have been working with and exposed to pesticides for many years (average 25 years (SD 14.4 years) and do not represent a naïve population for this acute exposure study. In addition, in the case of the cypermethrin sprayers, some of the exposures may have come from home where pyrethroid insecticides are widely used.

Nevertheless, this study is novel in that it examined whether acute exposures to pesticides is associated with acute changes in thyroid hormone levels by measuring pesticide metabolites and thyroid hormones over a 2-day period before and after farmers sprayed the targeted pesticides. Previous studies have focused on the chronic effects on the thyroid using a cross-sectional or longitudinal design using an un-exposed control group for comparison or looking an individual change over time (typically the growing season). The finding that acute exposures can acutely alter thyroid hormone levels suggests a mechanism for the development of chronic HPT axis disruption.

## 5. Conclusions

In studies of more chronic human exposures to pesticides, there is growing evidence that some pesticides can act as endocrine disrupters, altering hypothalamic–pituitary–thyroid (HPT) axis hormone levels. However, it was not clear if this disruption from chronic and repeated exposures can also be seen with acute exposures. This paper demonstrates that increases of specific pesticide metabolites from the morning before spraying to the morning after spraying are associated with concurrent changes in hormone levels. A one μg/g creatinine increase in urinary TCP significantly decreases FT3 and T3 across the same time period; a one-unit increase in urinary *cis*, *trans*-DCCS significantly increases T4. For herbicide spraying, the increase in one unit of urinary paraquat from the morning before to the morning after spraying significantly reduceds FT3 and T3, and the increase in one unit of urinary glyphosate significantly increased T4. These findings suggest that repeated acute exposures to pesticides could result in chronic disequilibrium of the HPT axis which may lead to associated metabolic disorders.

## Figures and Tables

**Table 1:** Demographic characteristics of conventional farmers (*n* = 78) spraying chlorpyrifos, cypermethrin, glyphosate or paraquat.

Variables	*n* (%)
Age	
Min-max	18–69
Mean (SD)	49.6 (12.3)
Sex	
Male	58 (74.4)
Female	20 (25.6)
Educational level	
Elementary	34 (44.2)
High school	42 (54.5)
Bachelor or higher	1 (1.3)
Marital status	
Single	10 (13.5)
Married	62 (83.8)
Widowed/divorced	2 (2.8)
Expense adequacy	
Enough for saving	6 (7.7)
Just enough	(46.2)
In debt	36 (46.2)
Agricultural work time (h/week)	
Mean (SD)	27.5 (9.5)
Have Second Job	
Number	40 (51.3)
Second job work time (h/week)	
Mean (SD)	20.9 (10.3)

**Table 2:** Risk factors and spraying factors of conventional farmers (*n* = 78) spraying chlorpyrifos, cypermethrin, paraquat and glyphosate.

Risk Factors	*n* (%)
Alcohol intake	
Current drinker	52 (66.7)
Not current drinker	26 (33.3)
Smoking	
Current smoker	15 (19.2)
Not current smoker	63 (80.8)
Heavy exercise in past month	
Yes	25 (32.1)
No	53 (67.9)
Stress in past 2–4 weeks	
Yes	60 (76.9)
Almost Never	18 (23.1)
Years of pesticide use	
Mean (SD)	25.0 (14.4)
Living near farm (1 km)	
Yes	74 (94.9)
No	4 (5.1)
Insecticide use in home	
Yes	77 (98.7)
No	1 (1.3)
Types of crop	
Rice	65 (83.3)
Sugarcane	77 (98.7)
others	41 (52.6)
Other parameters	Mean (SD)
Triglyceride	147.6 (122.5)
BMI (kg/m^2^)	25.5 (6.4)
Amount of time spraying (min)	
Chlorpyrifos	37.8 (21.5)
Cypermethrin	49.3 (37.7)
Paraquat	61.0 (31.1)
Glyphosate	37.4 (20.8)
Volume of pesticide spray (L)	
Chlorpyrifos	88.4 (81.3)
Cypermethrin	194.0 (257.2)
Paraquat	394.8 (392.1)
Glyphosate	79.6 (59.1)
Size of area sprayed (Rai)	
Chlorpyrifos	2.9 (2.1)
Cypermethrin	5.3 (3.8)
Paraquat	5.9 (3.5)
Glyphosate	2.2 (1.5)

**Table 3:** Urinary metabolite concentrations at three time points for pesticide sprayers: morning void urine the day before spraying, end of spraying activity, morning void urine the day after spraying.

Urinary Metabolites	Sprayed Chlorpyrifos		Sprayed Cypermethrin		
	TCP (*n* = 51) (µg/g Creatinine)	TDEP (*n* = 47) (nmol/g Creatinine)	3PBA (*n* = 47) (nmol/g Creatinine)	DCCA (*n* = 47) (nmol/g Creatinine)	TCyper (*n* =47) (nmol/g Creatinine)
First morning urine on day before spraying					
Detection frequency (%)	51 (100)	47 (100)	47 (100)	32 (68.1)	47 (100)
(1) GM (GSD)	4.31 (2.93)	458.38 (3.48)	82.32 (1.87)	18.61 (2.17)	108.56 (1.73)
Range	0.4–139.8	54.1–6634.2	21.54–311.06	5.81–106.70	37.71–323.76
Urine at the end of spraying event					
Detection frequency (%)	51 (100)	47 (100)	47 (100)	39 (83.0)	47 (100)
(2) GM (GSD)	7.79 (3.39)	346.99 (4.05)	149.35 (2.02)	29.12 (2.01)	190.01 (1.81)
Range	0.3–164.0	21.5–7044.5	43.38–533.79	4.53–129.02	58.56–584.06
First morning urine the next day after spraying					
Detection frequency (%)	51 (100)	47 (100)	47 (100)	35 (74.5)	47 (100)
(3) GM (GSD)	14.06 (2.34)	540.61 (3.10)	169.93 (1.88)	30.53 (2.42)	212.98 (1.76)
Range	2.41–141.20	51.42–7863.60	49.90–828.82	5.70–162.39	75.19–992.27
Paired comparisons of time points					
*p* value from repeated measures ANOVA on ln values	(1)–(2) *p* = 0.002	(1)–(2) *p* = 0.234	(1)–(2) *p* < 0.001	(1)–(2) *p* < 0.001	(1)–(2) *p* < 0.001
	(1)–(3) *p* < 0.001	(1)–(3) *p* = 0.421	(1)–(3) *p* < 0.001	(1)–(3) *p* < 0.001	(1)–(3) *p* < 0.001
	(2)–(3) *p* < 0.001	(2)–(3) *p* = 0.008	(2)–(3) *p* = 0.108	(2)–(3) *p* = 0.710	(2)–(3) *p* = 0.132

TCP = Specific metabolite of chlorpyrifos; TDEP = DEP + DETP; TCyper = 3PBA + DCCA.

**Table 4:** Urinary metabolite concentrations of paraquat and glyphosate at three time points for pesticide sprayers: morning void urine the day before spraying, end of spraying activity, morning void urine the day after spraying.

Parameter	Paraquat (*n* = 51) (µg/g Creatinine)	Glyphosate (*n* = 48) (µg/g Creatinine)
First morning urine on day before spraying		
Detection frequency (%)	32 (62.7)	48 (100)
(1) GM (GSD)	1.80 (2.74)	23.36 (2.61)
Range	0.30–10.68	3.05–138.07
Urine at the end of spraying event		
Detection frequency (%)	48 (94.1)	48 (100)
(2) GM (GSD)	5.96 (2.59)	39.90 (2.54)
Range	0.34–46.51	6.15–195.62
First morning urine the next day after spraying		
Detection frequency (%)	39 (76.5)	48 (100)
(3) GM (GSD)	2.99 (3.09)	48.47 (2.62)
Range	0.41–124.2	5.02–321.56
Paired comparison of time points		
*p* value from repeated measures ANOVA on ln values	(1)–(2) *p* < 0.001	(1)–(2) *p* < 0.001
	(1)–(3) *p* = 0.016	(1)–(3) *p* < 0.001
	(2)–(3) *p* = 0.001	(2)–(3) *p* = 0.194

**Table 5:** Comparison between thyroid hormones on the day before and the day after spraying insecticides chlorpyrifos and cypermethrin.

Thyroid Hormone		Sprayed Chlorpyrifos (*n* = 51)			Sprayed Cypermethrin (*n* = 48)		
		Before Spraying	After Spraying	*p*-Value ^a^	Before Spraying	After Spraying	*p*-Value ^a^
TSH (µIU/mL)	GM (GSD) Range	1.44 (1.99) 0.15–8.6	1.51 (1.76) 0.32–7.88	0.551	1.55 (1.66) 0.53–7.85	1.51 (1.99) 0.15–5.87	0.660
FT3 (ng/dL)	GM (GSD) Range	0.29 (1.18) 0.21–0.65	0.30 (1.18) 0.20–0.60	0.056	0.29 (1.15) 0.19–0.40	0.29 (1.12) 0.22–0.41	0.625
FT4 (ng/dL)	GM (GSD) Range	0.92 (1.17) 0.7–1.33	0.97 (1.17) 0.7–1.51	0.002*	0.99 (1.16) 0.71–1.34	0.99 (1.12) 0.80–1.39	0.990
T3 (µg/dL)	GM (GSD) Range	0.90 (1.23) 0.59–1.88	0.86 (1.24) 0.57–1.43	0.073	0.84 (1.27) 0.51–1.62	0.86 (1.24) 0.53–1.45	0.541
T4 (µg/dL)	GM (GSD) Range	8.14 (1.22) 4.8–12.43	7.94 (1.24) 4.55–12.35	0.265	8.05 (1.18) 5.47–11.25	7.82 (1.19) 5.10–12.06	0.069

^a^*t*-test on ln of paired thyroid hormone levels before spraying to after spraying; * = Significant at *p* < 0.05.

**Table 6:** Comparison between thyroid hormones on the day before and the day after spraying herbicides paraquat and glyphosate.

Thyroid Hormone		Sprayed Paraquat (*n* = 51)			Sprayed Glyphosate (*n* = 48)		
		Before Spraying	After Spraying	*p*-Value ^a^	Before Spraying	After Spraying	*p*-Value ^a^
TSH (µIU/mL)	GM (GSD) Range	1.30 (2.07) 0.16–6.4	1.21 (1.90) 0.23–6.48	0.490	1.30 (2.07) 0.14–4.44	1.21 (1.90) 0.14–6.94	0.426
FT3 (ng/dL)	GM (GSD) Range	0.30 (1.14) 0.24–0.44	0.31 (1.12) 0.23–0.39	0.203	0.30 (0.05) 0.21–0.39	0.30 (0.04) 0.22–0.40	0.674
FT4 (ng/dL)	GM (GSD) Range	0.95 (1.16) 0.75–1.44	0.96 (1.15) 0.76–1.4	0.357	1.00 (0.18) 0.65–1.55	0.98 (0.17) 0.67–1.46	0.409
T3 (µg/dL)	GM (GSD) Range	0.89 (1.26) 0.56–1.74	0.93 (1.24) 0.58–1.48	0.078	0.89 (0.20) 0.39–1.26	0.89 (0.15) 0.59–1.26	0.978
T4 (µg/dL)	GM (GSD) Range	7.99 (1.20) 5.16–11.86	7.80 (1.17) 5.51–11.32	0.274	7.75 (1.33) 5.16–11.30	7.99 (1.58) 5.18–11.27	0.202

^a^*t*-test on ln of paired thyroid hormone levels before spraying to after spraying; * = Significant at *p* < 0.05.

**Table 7:** Model for change in thyroid hormone levels (logged morning after spraying—logged morning before spraying) as a function of the change in urinary metabolite levels for sprayed insecticide chorpyrifos (logged morning after spraying—logged morning before spraying).

ΔLNthyroid Hormones	ΔLNTCP (µg/g Creatinine)			ΔLNTDEP (nmol/g Creatinine)		
	B	Standard Error	*p*-Value	B	Standard Error	*p*-Value
ΔLNTSH (nIU/mL)	1.2	78.6	0.988	41.1	64.1	0.521
ΔLNFT3 (pg/dL)	−27.0	12.7	0.033 *	−0.16	12.7	0.990
ΔLNFT4 (pg/dL)	9.1	14.3	0.526	14.5	9.5	0.128
ΔLNT3 (ng/dL)	−46.6	17.1	0.007 *	23.6	13.4	0.077
ΔLNT4 (ng/dL)	26.1	17.6	0.139	−9.0	17.8	0.612

ΔLNTCP model controlled for gender, stress (y/n) and triglyceride level; TDEP = DEP + DETP; ΔLNTDEP controlled for gender, education, smoking(y/n), stress (y/n) and triglyceride; Δ = Different between the day after—before values; * *F*-test significant at *p* < 0.05.

**Table 8:** Model for change in thyroid hormone levels (logged morning after spraying—logged morning before spraying) as a function of the change in urinary metabolite levels for sprayed insecticide cypermethrin (logged morning after spraying—logged morning before spraying).

Δthyroid Hormone	ΔLN3PBA ^a^ (nmol/g Creatinine)			ΔLNcis, *trans*-DCCA ^b^ (nmol/g Creatinine)			ΔLNTCyper ^c^ (nmol/g Creatinine)		
	B	Standard Error	*p*-Value	B	Standard Error	*p*-Value	B	Standard Error	*p*-Value
ΔLNTSH (nIU/mL)	43.2	88.9	0.627	47.9	56.6	0.397	65.8	94.7	0.487
ΔLNFT3 (pg/dL)	24.3	18.3	0.185	26.4	17.1	0.122	35.1	19.2	0.067
ΔLNFT4 (pg/dL)	2.7	28.8	0.925	13.6	22.2	0.539	11.7	32.5	0.718
ΔLNT3 (ng/dL)	−4.1	41.7	0.921	17.7	25.8	0.492	4.3	46.3	0.926
ΔLNT4 (ng/dL)	−18.1	22.9	0.431	34.8	17.5	0.046 *	−1.1	22.5	0.962

^a,c^ Control for gender, stress (y/n), education level and triglyceride level; ^b^ Control for age, gender, stress(y/n), education level and triglyceride level; TCyper = 3PBA + DCCA; Δ = Different between the day after—before values; * *F*-test significant at *p* < 0.05.

**Table 9:** Model for change in thyroid hormone levels (logged morning after spraying—logged morning before spraying) as a function of the change in urinary metabolite levels for sprayed herbicides paraquat or glyphosate (logged morning after spraying—logged morning before spraying).

Δthyroid Hormone	ΔLNparaquat ^a^ (µg/g Creatinine)			ΔLNglyphosate ^b^ (µg/g Creatinine)		
	B	Standard Error	*p*-Value	B	Standard Error	*p*-Value
ΔLNTSH (nIU/mL)	29.5	60.6	0.627	68.1	51.1	0.183
ΔLNFT3 (pg/dL)	−30.0	14.3	0.036 *	1.7	12.1	0.888
ΔLNFT4 (pg/dL)	−7.0	10.2	0.493	11.1	10.9	0.311
ΔLNT3 (ng/dL)	−38.1	18.2	0.036 *	11.7	25.1	0.642
ΔLNT4 (ng/dL)	9.4	15.4	0.539	24.5	12.2	0.045 *

^a^ controlled for gender, debt (y/n), current smoking (y/n); ^b^ controlled for gender; Δ = Different between the day—before values; * *F*-test significant at *p* < 0.05.

## Data Availability

The data presented in this study are available on request from the corresponding author. The data are not publicly available due to privacy of subjects.
